# Population Pharmacokinetics and Dosing Optimization of Piperacillin-Tazobactam in Critically Ill Patients on Extracorporeal Membrane Oxygenation and the Influence of Concomitant Renal Replacement Therapy

**DOI:** 10.1128/Spectrum.00633-21

**Published:** 2021-12-22

**Authors:** Jongsung Hahn, Kyoung Lok Min, Soyoung Kang, Seungwon Yang, Min Soo Park, Jin Wi, Min Jung Chang

**Affiliations:** a Department of Pharmacy and Yonsei Institute of Pharmaceutical Sciences, College of Pharmacy, Yonsei University, Incheon, Republic of Korea; b Department of Pharmaceutical Medicine and Regulatory Sciences, Colleges of Medicine and Pharmacy, Yonsei University, Incheon, Republic of Korea; c Department of Clinical Pharmacology, Severance Hospital, Yonsei University College of Medicine, Seoul, Republic of Korea; d Department of Pediatrics, Yonsei University College of Medicine, Seoul, Republic of Korea; e Division of Cardiology, Department of Internal Medicine, Gachon University Gil Medical Center, Incheon, Republic of Korea; f Division of Cardiology, Department of Internal Medicine, Yonsei University College of Medicine, Seoul, Republic of Korea; INTHERES

**Keywords:** PTA, continuous renal replacement therapy, creatinine clearance, extracorporeal membrane oxygenation, pharmacodynamics, pharmacokinetics, piperacillin, tazobactam

## Abstract

Critical illness and extracorporeal circulation, such as extracorporeal membrane oxygenation (ECMO) and continuous renal replacement therapy (CRRT), may alter the pharmacokinetics of piperacillin-tazobactam. We aimed to develop a population pharmacokinetic model of piperacillin-tazobactam in critically ill patients during ECMO or CRRT and investigate the optimal dosage regimen needed to achieve ≥90% of patients attaining the piperacillin pharmacodynamic target of 100% of dosage time above MIC of 16 mg/L. This prospective observational study included 26 ECMO patients, of which 13 patients received continuous venovenous hemodiafiltration (CVVHDF). A population pharmacokinetic model was developed using nonlinear mixed-effects models, and Monte Carlo simulations were performed to evaluate creatinine clearance (CrCL) and infusion method in relation to the probability of target attainment (PTA) in four patient groups according to combination of ECMO and CVVHDF. A total of 244 plasma samples were collected. In a two-compartment model, clearance decreased during ECMO and CVVHDF contributed to an increase in the volume of distribution. The range of PTA reduction as CrCL increased was greater in the order of intermittent bolus, extended infusion, and continuous infusion method. Continuous infusion should be considered in critically ill patients with CrCL of ≥60 mL/min, and at least 12, 16, and 20 g/day was required for CrCL of <40, 40 to 60, and 60 to 90 mL/min, respectively, regardless of ECMO or CVVHDF. In patients with CrCL of ≥90 mL/min, even a continuous infusion of 24 g/day was insufficient to achieve adequate PTA. Therefore, further research on permissible high continuous infusion dose focused on the risk of toxicity is required. (This trial has been registered at ClinicalTrials.gov under registration no. NCT02581280, December 1, 2014.)

**IMPORTANCE** To the best of our knowledge, this is the first large prospective pharmacokinetic/pharmacodynamic (PK/PD) study of piperacillin-tazobactam in ECMO patients. We used piperacillin-tazobactam plasma concentration data from four different cases (concomitant use of ECMO and CVVHDF, receiving ECMO only, weaned from ECMO and receiving CVVHDF, and weaned from ECMO and not receiving CVVHDF) to provide preliminary insights into the incremental effects of critical illness, ECMO, and CVVHDF on PK. Our analysis revealed that volume of distribution increased in patients on CVVHDF and clearance decreased during ECMO and as creatinine clearance was reduced. When targeting 100% *f*T_>MIC_ (16 mg/L, clinical breakpoint for Pseudomonas aeruginosa), continuous infusions would have achieved the highest percentage of target attainment compared to intermittent bolus or extended infusion if the total daily dose was the same. Continuous infusion should be considered in critically ill patients with creatinine clearance of ≥60 mL/min, regardless of ECMO or CVVHDF.

## INTRODUCTION

Extracorporeal membrane oxygenation (ECMO) is mechanical circulatory support for the bridge to recovery or transplantation in patients with refractory cardiopulmonary failure, and its use has increased in recent years (1). More than 60% of adult patients receiving ECMO develop nosocomial infections, which are related to the multiple invasive devices required for management, long-term mechanical ventilation, and hospital stays (2). The infectious complications of ECMO increase the risk of death by 38% to 63% (3, 4); therefore, prevention and treatment of infections are important.

Piperacillin-tazobactam is an intravenous β-lactam antibiotic commonly used for the empirical or directed treatment of infections in critically ill patients, including ECMO patients (5). Piperacillin exhibits activity against a broad range of Gram-negative organisms, including Escherichia coli, Klebsiella pneumonia, and Pseudomonas aeruginosa, and shows high tolerability (6). For piperacillin-tazobactam, which has a time-dependent activity pattern, the pharmacodynamic (PD) target is related to the percentage of time that the unbound drug concentration remains above the MIC (% *f*T_>MIC_) (7). The optimal PD target for piperacillin exposure and clinical effect is still controversial (8). In general, the *f*T_>MIC_ required for optimal bactericidal activity for piperacillin has been reported to be 50% in mild/moderate infections (9, 10). However, recent clinical data suggest that a longer exposure of 100% might be required for critically ill patients (11, 12).

Piperacillin-tazobactam is a moderate protein binding (30%) and hydrophilic drug, with a 71% recovery from *ex vivo* ECMO circuits after 48 h (13). Critical illness and ECMO itself can significantly alter the pharmacokinetics (PK) of piperacillin-tazobactam, which can result in subtherapeutic levels or drug accumulation associated with clinical failure. The use of continuous renal replacement therapy (CRRT) also complicates the PK of piperacillin-tazobactam (14). In patients undergoing CRRT, piperacillin clearance was varied and lower than that of healthy volunteers. It might be because of renal impairment, which leads to requirement of CRRT. Piperacillin volume of distribution was also higher in CRRT patients, which was due to use of fluid resuscitation and pathophysiological changes caused by critical illness (12).

However, to date, studies on the PK of piperacillin-tazobactam have enrolled only patients receiving ECMO or CRRT separately. In this study, we developed a population PK model of piperacillin-tazobactam in ECMO patients and performed Monte Carlo simulations to evaluate various clinical covariates, MIC levels, and dosing regimens in relation to *f*T_>MIC_ and finally suggested an optimal dosage regimen to achieve the PD target of 100% *f*T_>MIC_ (16 mg/L).

## RESULTS

### Study population.

The study included 26 ECMO patients, of which 19 (73.1%) were male; the median age was 57 years (range, 20 to 89), and the median body weight was 70 kg (range, 40.8 to 92.5). Thirteen patients (50%) underwent continuous venovenous hemodiafiltration (CVVHDF). All 26 patients provided samples for PK analysis while they were on ECMO. Among them, 14 patients who were weaned from ECMO provided samples to explore the effect of ECMO on piperacillin-tazobactam PK. A total of 244 piperacillin-tazobactam plasma concentration measurements were available: 67 during concomitant use of ECMO and CVVHDF, 96 during ECMO only, and 81 after ECMO discontinuation (27 on CVVHDF and 54 not on CVVHDF). The demographic characteristics of the patients are presented in Table 1.

**TABLE 1:** Demographic characteristics

Characteristic	N (%) or median [range] (*n* = 26)^*a*^
Age (yrs)	57 [20–89]
Male	19 (73.1)
Body wt (kg)	70 [40.8–92.5]
Body mass index (kg/m^2^)	26 [18.1–31.3]
APACHE II	32 [6–46]
Indication for VA-ECMO	
Cardiomyopathy	3
Valvular heart disease	4
ST-elevation myocardial infarction	16
Non-ST-elevation myocardial infarction	3
Duration of VA-ECMO (h)	135 [52.1–264]
ECMO flow rate (L/min)	3.0 [0.3–4.1]
Use of continuous CVVHDF	13
Blood flow rate (mL/min)	150 [100–160]
Dialysate flow rate (mL/h)	1200 [800–1400]
Blood chemistry, serum levels	
Total plasma protein (g/dL)	4.9 [2.7–6.8]
Albumin (g/dL)	2.9 [1.4–3.7]
Total bilirubin (mg/dL)	2.1 [0.5–8.1]
Blood urea nitrogen (mg/dL)	23.1 [7–64.2]
Serum creatinine (mg/dL)	1.4 [0.37–5.22]
Lactate (mmol/L)	1.6 [0.8–20.0]
Partial pressure of carbon dioxide (mm Hg)	29.1 [13.5–46.7]
Tympanic body temp (°C)	36.9 [33–38.7]
CrCL^*b*^ (mL/min)	54.7 [16.2-157]
CrCL^*b*^ when receiving CVVHDF (mL/min)	40.5 [18.0–111]
CrCL^*b*^ when not receiving CVVHDF (mL/min)	63.2 [16.2–157]
Piperacillin-tazobactam dosage (intermittent bolus)	
2/0.25 g every 6 hours (q6h)	9
3/0.375 g q6h	4
4/0.5 g q6h	9
4/0.5 g q8h	4

a Data are presented as numbers (percentages) or medians [ranges].

b Creatinine clearance estimated using the Cockroft–Gault equation. APACHE II, acute physiology and chronic health evaluation II; CVVHDF, continuous venovenous hemodiafiltration; VA-ECMO, venoarterial extracorporeal membrane oxygenation therapy.

### Population PK analysis.

A two-compartment PK model with first-order linear elimination and proportional residual variability best described the observed concentration data for piperacillin. Interindividual variability (IIV) was included for clearance (CL), central volume of distribution (V1), and peripheral volume of distribution (V2). The inclusion of creatinine clearance (CrCL) calculated by the Cockcroft-Gault equation (15) as a covariate on clearance significantly improved the model (change in objective function value [ΔOFV] of −23.1). The influence of serum creatinine, blood urea nitrogen, and glomerular filtration rate was also tested but showed a less significant impact. The full model supported the inclusion of CrCL and the presence of ECMO as covariates for CL and total bilirubin and the presence of CVVHDF as covariates for V1. In backward elimination, extracting the total bilirubin from V1 increased the OFV to <6.63 (*P* > 0.01) (Table S1). The final PK model is described as follows:

CL=9.4×(1−0.092×ECMO)+[0.115×(CrCL−54.7)]

V1=6.56×(1+1.46×CVVHDF)

V2=14.2

Q=17.2

A two-compartment PK model with first-order linear elimination and combined residual variability was the best fit for the tazobactam concentration-time data. IIV was included for CL and V1. After forward selection and backward elimination, CrCL and the presence of ECMO were identified as significant covariates of CL (Table S2). The final PK model is described as follows:

CL(L/h)=7.93×e(−0.0723×ECMO)+0.104×(CrCL−54.7)

V1(L)=8.58

V2(L)=10.5

Q(L/h)=17.1

The population parameters of the final models for piperacillin and tazobactam are presented in Tables 2 and 3, respectively. All estimates were within the 95% confidence intervals (CIs) obtained from 5,000 bootstrap runs. The goodness of fit (GOF) plots for piperacillin and tazobactam are presented in Fig. S1 and S2, respectively. The individual-fitted and population-fitted concentrations were unbiased and did not show any model misspecification. The prediction-corrected visual predictive check (pc-VPC) plot revealed that the 5th to 95th percentiles of the predicted data overlaid most of the observed data, indicating good predictive power (Fig. S3 and S4). These results confirmed that the final model was sufficiently robust to simulate concentration-time profiles.

**TABLE 2:** Piperacillin bootstrap results

Parameter	Structural model (RSE %)^*a*^ [shrinkage%]	Final model data		
		Final model (RSE %) [shrinkage %]	Bootstrap (5,000 replicates)	
			Median	95% CI (2.5%–97.5%)
Fixed effects				
Θ*_CL_*	7.9 (11)	9.4 (7)	9.601	8.307, 11.43
Θ*_V_*_1_	11.1 (23)	6.56 (18)	7.824	3.038, 14.52
Θ*_V_*_2_	13.3 (19)	14.2 (14)	12.70	9.095, 17.04
Θ_Q_	16.3 (32)	17.2 (22)	14.53	7.318, 23.92
Θ*_ECMO_*		–0.092 (33)	–0.099	–0.170, –0.020
Θ*_CrCL_*		0.115 (17)	0.120	0.051, 0.166
Θ*_CVVHDF_*		1.46 (40)	1.837	0.076, 5.257
Random effects				
Interindividual variability				
*ω_CL_*^2^	0.276 (20) [1]	0.0523 (33) [8]	0.0544	0.017, 0.129
*ω_V_*_1_^2^	0.313 (49) [32]	0.291 (65) [31]	0.431	0.083, 1.279
*ω_V_*_2_^2^	0.0898 (51) [45]	0.138 (34) [28]	0.115	0.013, 0.325
Residual variability				
*σ^2^_proportional_*	0.107 (26) [7]	0.0979 (20) [8]	0.0874	0.055, 0.126

a RSE %, relative standard error; RSE % = (standard error/parameter estimate) × 100; CI, confidence interval; CL, systemic clearance; V1, central volume of distribution; V2, peripheral volume of distribution; Q, intercompartmental clearance; ECMO, extracorporeal membrane oxygenation therapy; CrCL, creatinine clearance; CVVHDF, continuous venovenous hemodiafiltration.

**TABLE 3:** Tazobactam bootstrap results

Parameter	Structural model (RSE%)^*a*^ [shrinkage%]	Final model		
		Final model (RSE%) [shrinkage%]	Bootstrap (5,000 replicates)	
			Median	95% CI (2.5%–97.5%)
Fixed effects				
Θ*_CL_*	6.68 (14)	7.93 (6)	7.71	6.50, 8.91
Θ*_V_*_1_	9.27 (32)	8.58 (12)	8.45	5.26, 13.92
Θ*_V_*_2_	10.9 (11)	10.5 (7)	10.3	8.75, 12.40
Θ_Q_	15.7 (24)	17.1 (14)	15.9	10.42, 28.04
Θ*_ECMO_*		–0.0723 (43)	–0.0722	–0.175, –0.010
Θ*_CrCL_*		0.104 (13)	0.0979	0.018, 0.121
Random effects				
Interindividual variability				
ω*_CL_*^2^	0.357 (21) [1]	0.0724 (40) [5]	0.0786	0.0250, 0.2997
*ω_V_*_1_^2^	0.509 (60) [27]	0.705 (61) [18]	0.721	0.281, 1.287
Residual variability				
*σ^2^_proportional_*	0.1 (38) [7]	0.0675 (35) [8]	0.0523	0.0184, 0.1082
*σ^2^_additional_*	0.364 (79) [7]	0.517 (56) [8]	0.4722	0.0950, 3.470

a RSE %, relative standard error, equal to (standard error/parameter estimate) × 100; CI, confidence interval; CL, systemic clearance; V1, central volume of distribution; V2, peripheral volume of distribution; Q, intercompartmental clearance; ECMO, extracorporeal membrane oxygenation; CrCL, creatinine clearance.

### Monte Carlo simulations.

**FIG 1:**
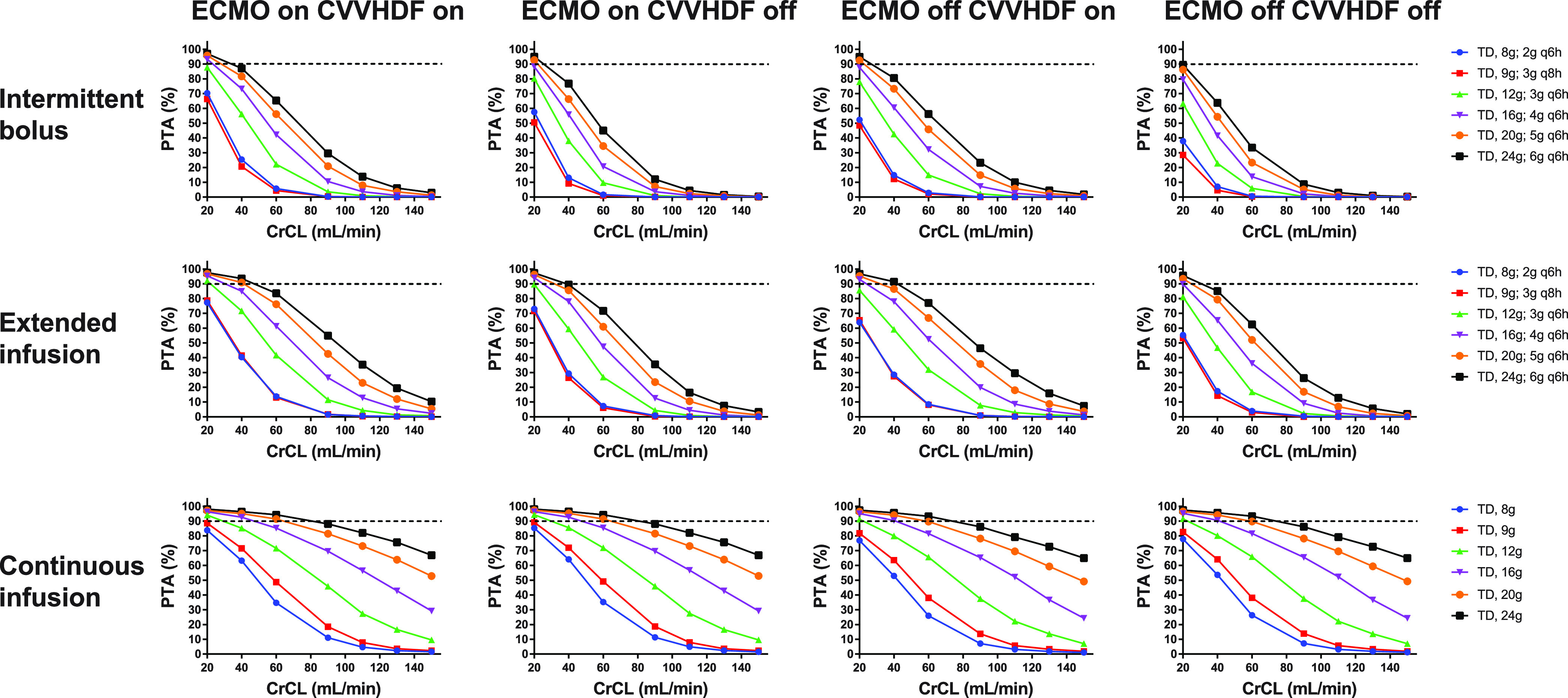
Probability of target attainment (100% *f*T_>MIC_ of 16 mg/L) for piperacillin in critically ill patients stratified by the presence of ECMO and CVVHDF at different CrCL levels when administered an intermittent bolus (upper panel), an extended infusion (middle panel), and a continuous infusion (lower panel). The dashed horizontal line indicates 90% PTA. CVVHDF, continuous venovenous hemodiafiltration; ECMO, extracorporeal membrane oxygenation; CrCL, creatinine clearance; PTA, probability of target attainment; TD, total daily dose.

The piperacillin PTA for 100% *f*T_>MIC_ (16 mg/L) of various dosing regimens in four patient groups (group 1 ECMO on CVVHDF on, group 2 ECMO on CVVHDF off, group 3 ECMO off CVVHDF on, and group 4 ECMO off CVVHDF off) with various CrCL levels at steady state (72 h of therapy) is shown in Fig. 1 and Table S3. The strong relationship between CrCL and piperacillin clearance is directly reflected in the PTA. The range of PTA reduction as CrCL increased was greater in the order of intermittent bolus, extended infusion, and continuous infusion. Of the simulated infusion methods, continuous infusions would have achieved the highest PTA across all groups compared to intermittent bolus or extended infusion if the total daily dose was the same. The calculated PTA was higher in patients who received ECMO than in those who weaned from ECMO. When administered by intermittent bolus or extended infusion, calculated PTA was higher in patients on CVVHDF than in those not on CVVHDF. However, in the case of continuous infusion, CVVHDF did not much affect PTA. The highest approved dose, 4/0.5 g of piperacillin/tazobactam every 6 h infused over 30 min, would have met the PD target for only 42.2% in group 1 (ECMO on CVVHDF), 20.7% in group 2 (ECMO on CVVHDF off), 32.2% in group 3 (ECMO off CVVHDF on), and 13.8% in group 4 (ECMO off CVVHDF off) in CrCL level of 60 mg/dL.

**FIG 2:**
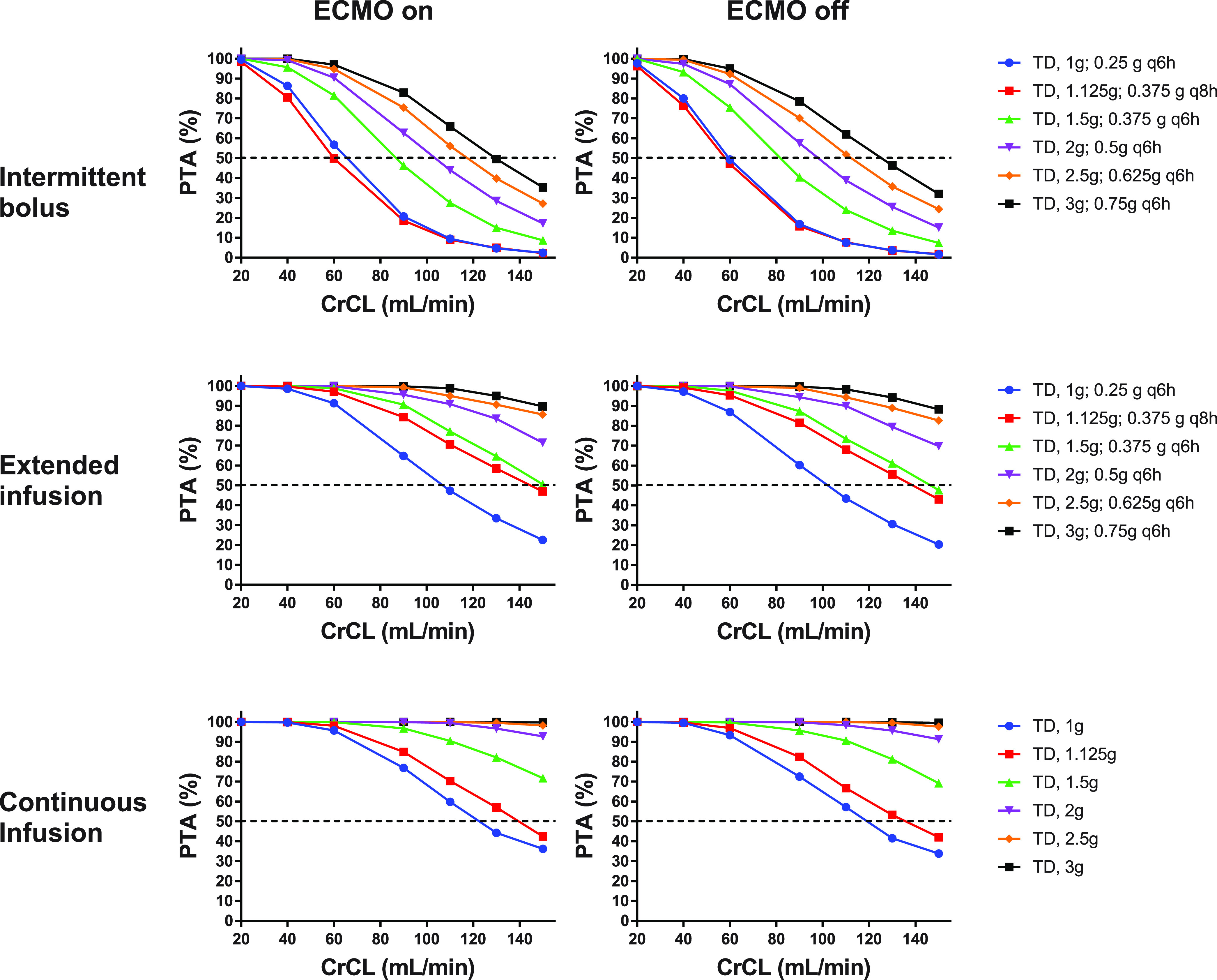
Probability of target attainment (63% *f*T > 2 mg/L) for tazobactam in critically ill patients stratified by the presence of ECMO at different CrCL levels when administered an intermittent bolus (upper panel), an extended infusion (middle panel), and a continuous infusion (lower panel). The dashed horizontal line indicates 50% PTA. ECMO, extracorporeal membrane oxygenation; CrCL, creatinine clearance; PTA, probability of target attainment; TD, total daily dose.

The tazobactam PTA of 63% *f*T >2 mg/L was also calculated (Fig. 2). Simulations suggested that PTA is above 50% with continuous infusion of 1.5 g/day regardless of ECMO and CrCL level.

**FIG 3:**
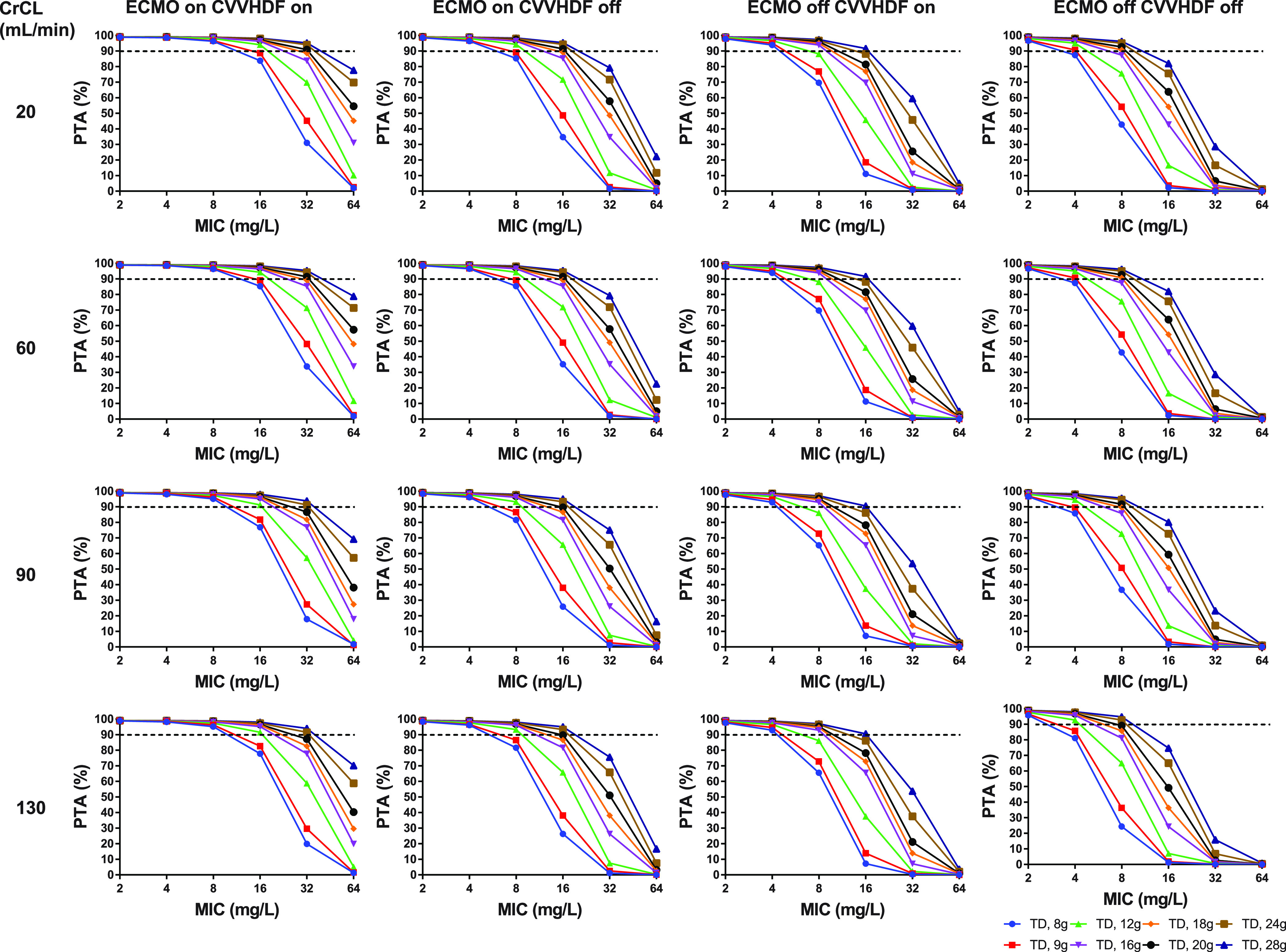
Probability of target attainment (100% *f*T_>MIC_ of 2, 4, 8, 16, 32, and 64 mg/L) for piperacillin in critically ill patients stratified by the presence of ECMO and CVVHDF at different CrCL levels (first column, CrCL 20 mL/min; second column, CrCL 60 mL/min; third column, CrCL 90 mL/min; last column, CrCL 130 mL/min) when administered as continuous infusion. The dashed horizontal line indicates 90% PTA. ECMO, extracorporeal membrane oxygenation; CrCL, creatinine clearance; CVVHDF, continuous venovenous hemodiafiltration; PTA, probability of target attainment; TD, total daily dose.

The piperacillin PTAs (100% *f*T_>MIC_) against MICs of 2 to 64 mg/L according to four CrCL levels (20, 60, 90, and 130 mL/min) in four groups are presented in Fig. 3. Eight dosage regimens comprising only the continuous infusion (8, 9, 12, 16, 18, 20, 24, and 28 g/day) were evaluated.

## DISCUSSION

To the best of our knowledge, this is the first large prospective pharmacokinetic/pharmacodynamic (PK/PD) study of piperacillin-tazobactam in ECMO patients. Two case-control studies showed that the PK of piperacillin-tazobactam did not differ significantly between critically ill patients receiving ECMO and those not receiving ECMO; however, this conclusion was reached by simple PK analysis using therapeutic drug monitoring results (16) or PK modeling that explored ECMO only as a covariate (17). In the current study, we used piperacillin-tazobactam plasma concentration data from four different cases (concomitant use of ECMO and CVVHDF, receiving ECMO only, weaned from ECMO and receiving CVVHDF, and weaned from ECMO and not receiving CVVHDF) to provide preliminary insights into the incremental effects of critical illness, ECMO, and CRRT on piperacillin-tazobactam PK. This could reflect a balance between independent changes in the volume of distribution and clearance in the presence of various clinical factors. Furthermore, we proposed an empirical piperacillin-tazobactam dosage regimen for ECMO patients based on ≥90% PTA for 100% *f*T_>MIC_ of 16 mg/L.

The PK of piperacillin in our study was best described by two-compartment models with linear elimination with a CL of 8.54 L/h or 9.4 L/h (receiving ECMO or weaned from ECMO, respectively; standardized CrCL of 54.7 mL/min), V1 of 16.14 L or 6.56 L (receiving CVVHDF or not receiving CVVHDF, respectively), V2 of 14.2 L, and Q of 17.2 L/h. Our final model showed that CL decreased in patients who received ECMO and V1 increased in patients on CVVHDF compared with those in patients who did not receive ECMO or were not on CVVHDF, respectively.

For ECMO, despite initial concerns about drug adsorption in the circuit, this sequestration does not appear to occur significantly with piperacillin and tazobactam, which are hydrophilic and have moderate protein binding. Instead, decreased clearance during ECMO may reflect a relatively higher severity of illness in patients receiving ECMO.

Piperacillin-tazobactam is eliminated predominantly through renal excretion, and its clearance is usually dependent on renal function and CRRT (18, 19). Interestingly, the inclusion of CVVHDF on clearance did not improve model performance. Previous studies did not yield conclusive data on the effect of CRRT on piperacillin-tazobactam PK, which might be attributed to patient baseline differences and variations in CRRT modality and intensity (12, 20, 21). Recent evidence has suggested that CRRT affects clearance only in patients with significantly impaired renal function (CrCL of ≤10 mL/min or urine output of <20 mL/h) (20, 22, 23). Since our patients on CVVHDF had residual renal function (median CrCL at the onset of CVVHDF, 34.4 mL/min), CVVHDF was found to be nonrelevant to CL. Instead, volume overload, which was the main cause of CVVHDF, might have contributed to the increased V1 of piperacillin. In addition, the CVVHDF membrane, polyacrylonitrile, could be responsible for the adsorption of piperacillin-tazobactam (24). This relationship of V1 with CVVHDF had a positive impact on the ability to achieve adequate PTA of piperacillin in patients on CVVHDF. Although maximum piperacillin concentration was lower due to high V1, minimum piperacillin concentrations were high, resulting in higher PTA in CVVHDF patients. In the final model, the CrCL calculated by the Cockcroft-Gault equation was included as a covariate on CL, which could indirectly reflect the clearing effect of CVVHDF.

Because treatments are often initiated without knowledge of culture-specific MIC data, the greatest susceptible MIC of 16 mg/L from EUCAST clinical breakpoint tables and 100% *f*T_>MIC_ were chosen for piperacillin empirical dosing (25).

Considering the possibility of toxicity, the lowest possible dose with frequent administration was chosen as the recommended dosage (Table 4). Tazobactam dosage was confirmed to be sufficient to achieve ≥50% PTA for 63% *f*T >2 mg/L. In patients with CrCL of ≥60 mL/min, continuous infusion should be considered because intermittent bolus or extended infusion could not achieve 90% PTA for piperacillin even at the highest dose of 24 g/day. Continuous infusion of at least 12, 16, and 20 g/day was required for CrCL of <40, 40 to 60, and 60 to 90 mL/min, respectively. Unfortunately, in patients with CrCL of ≥90 mL/min, continuous infusion of 24 g/day was insufficient to achieve adequate PTA. For the target MIC of 8 mg/L, which is the clinical breakpoint for *Enterobacteriaceae*, continuous infusion of 16 g/day granted optimal PTAs in most patients, but at least 20 g/day was required in patients weaned from ECMO and not receiving CVVHDF (Fig. 3).

**TABLE 4:** Proposed empirical piperacillin-tazobactam dosage regimen

Category of patients		Dosing regimen			
		ECMO on, CVVHDF on	ECMO on, CVVHDF off	ECMO off, CVVHDF on	ECMO off, CVVHDF off
CrCL (mL/min)	Infusion method				
40<	Intermittent bolus	5 g q6h–6 g q6h	5 g q6h–6 g q6h	5 g q6h–6 g q6h	6 g q6h^*a*^
	Extended infusion	3 g q6h–4 g q6h	4 g q6h–5 g q6h	4 g q6h–5 g q6h	4 g q6h–5 g q6h
	Continuous infusion	12 g/day	12 g/day	12 g/day	12 g/day
40–60	Extended infusion	5 g q6h–6 g q6h	6 g q6h^*a*^	6 g q6h^*a*^	Not recommended
	Continuous infusion	16 g/day–20 g/day	16 g/day–20 g/day	16 g/day–20 g/day	16 g/day–20 g/day
60–90	Continuous infusion	20 g/day–24 g/day	20 g/day–24 g/day	20 g/day–24 g/day	20 g/day–24 g/day
≥90	Continuous infusion	>24 g/day^*a*^	>24 g/day^*a*^	>24 g/day^*a*^	>24 g/day^*a*^

a This dosage regimen might not achieve adequate PTA despite the high total daily dose of 24 g/day. Dosing regimen suggestions were based on the achievement of 90% PTA (target: 100% *f*T_>MIC_ of 16 mg/liter) for piperacillin and 50% PTA (target: 63% *f*T > 2 mg/liter) for tazobactam. ECMO, extracorporeal membrane oxygenation; CVVHDF, continuous venovenous hemodiafiltration; CrCL, creatinine clearance.

This study has some limitations. First, this was a single-center study, and caution is advised against the extrapolation of the dosage regimen to any patient falling outside the characteristics of the studied cohort. Second, we calculated unbound concentrations based on previous studies, suggesting consistent protein binding kinetics in critically ill patients. Third, because of the absence of urine data, we could not explore measured CrCL or residual diuresis as a potential covariate of CL in our PK model. We did not measure pre- or postfilter concentrations or dialysate concentrations, and therefore we could not quantify CVVHDF clearance. Fourth, CVVHDF status was used as a binary covariate. We tested the CVVHDF duration, dialysate flow rate, and blood flow rate as covariates, but these did not improve the model. This may have occurred because a consistent setting of CVVHDF was applied to most of the study patients. Future studies should further investigate the effects of duration, mode, and intensity of CRRT on piperacillin-tazobactam PK during ECMO. Nevertheless, the major strengths of this study are its large sample size (26 patients) and a rich sampling scheme.

In conclusion, we revealed that piperacillin CL decreased during ECMO, reflecting a higher severity of illness, and V1 increased during CVVHDF due to fluid overload. The calculated PTA (100% *f*T_>MIC_ of 16 mg/L) increased in patients receiving ECMO and CVVHDF more than in those who did not receive those treatments. When ≥90% PTA is targeted as empirical dosing, continuous infusion should be considered in patients with CrCL of ≥60 mL/min regardless of ECMO or CVVHDF. However, in patients with CrCL of ≥90 mL/min, even 24 g/day was insufficient to achieve adequate PTA. Therefore, further research on permissible high continuous infusion dose focused on preventing the risk of toxicity is required.

## MATERIALS AND METHODS

### Study design and population.

This prospective observational PK/PD study was conducted at the cardiac intensive care unit (CCU) of Severance Cardiovascular Hospital in Seoul, South Korea between November 2015 and January 2019. This study was conducted in accordance with the Declaration of Helsinki and national and institutional standards. The study protocol was approved by the Institutional Review Board (IRB no. 4-2014-0919) of Severance Hospital. Written informed consent was obtained from each patient or the legal representative of an unconscious patient prior to enrollment. This study included critically ill patients aged 19 years or older who were admitted to the CCU for venoarterial (VA) ECMO and were prescribed piperacillin-tazobactam. The exclusion criterion was the use of drugs known to change the plasma concentrations of piperacillin-tazobactam.

### Details of ECMO and CRRT.

The ECMO circuit included a centrifugal blood pump with a pump controller (Capiox SP-101, Terumo Inc., Tokyo, Japan), an air-oxygen mixer (Sechrist Ind., Anaheim, CA, USA), and conduit tubing (Capiox EBS Circuit with X coating, Terumo Inc.).

Patients underwent independent CRRT using continuous venovenous hemodiafiltration (CVVHDF, Prismaflex; Baxter Inc., IL, USA). The Prismaflex ST100 set configurations were used with a polyacrylonitrile AN 69 membrane with a 1 m^2^ surface area. All CVVHDF settings were established at the discretion of the treating physician.

### Dosing and sampling procedure.

The standard empirical dose used at our institution for patients with normal renal function is 4/0.5 g of intravenous piperacillin-tazobactam every 6 h (q6h) or q8h infused over 40 min per dose. However, when the CrCL was <40 mL/min or the patient was receiving CRRT, the piperacillin-tazobactam dosage was reduced to 3/0.375 g or 2/0.25 g q6h.

Blood samples were collected from an existing arterial catheter just before piperacillin-tazobactam administration (time zero) and then 0 to 0.5, 0.5 to 1, 1 to 2, 2 to 4, 4 to 6, and 6 to 8 h afterward. The samples were collected between days 2 and 4 of the VA-ECMO. In the case of concomitant CRRT, the first sample was obtained at least 24 h after CRRT initiation. If the patient was able to be weaned from ECMO, the collection of blood samples for PK analysis was repeated on day 2 of ECMO discontinuation at 0 (predose), 0 to 0.5, 0.5 to 1, 1 to 2, 2 to 4, 4 to 6, and 6 to 8 h of piperacillin-tazobactam administration.

### Plasma concentration assay.

Blood samples were added to EDTA tubes and centrifuged at 1,500 × *g* for 15 min within 1 h of collection and stored at –80°C until analysis. Validated liquid chromatography-tandem mass spectrometry assays were used to quantify total piperacillin and tazobactam concentrations (26). The linear concentration ranges of piperacillin and tazobactam were 1 to 220 mg/L and 0.5 to 28 mg/L, respectively. The lower limit of quantification was 0.5 mg/L for both piperacillin and tazobactam. The intraassay accuracies of the quality control samples (1, 3, 30, and 160 mg/L) ranged from 94.9% to 96.5% for piperacillin and from 90.0% to 94.7% for tazobactam.

### Population PK analyses.

Population PK analyses were conducted using nonlinear mixed-effects modeling (NONMEM; version 7.4; ICON Development Solutions, Ellicott, MD, USA) and the first-order conditional estimation with interaction method. We explored one-, two-, and three-compartment models. Interindividual variability (IIV) was modeled exponentially, and additive, proportional, and combined residual error models were evaluated. Model selections were based on the decrease in objective function value (OFV), goodness of fit (GOF) plots, and relative standard errors of the parameters. When adding another parameter to the model, a decrease in OFV greater than 3.84 points between two nested models was significant at a *P* value of 0.05.

The relationships between individual PK parameter estimates and the covariates (sex, age, weight, total plasma protein, glomerular filtration rate, blood urea nitrogen, serum creatinine, CrCL estimated using the Cockroft–Gault equation, total bilirubin, use of CRRT, CRRT blood flow rate, CRRT dialysate flow rate, CRRT duration, use of ECMO, ECMO pump speed, ECMO flow rate, and ECMO duration) were investigated in scatterplots, and biologically plausible explanations for altering PKs were explored. Continuous covariates were centered on the median population value. Based on the χ^2^ test, stepwise covariate modeling with forward inclusion (*P* < 0.05, OFV decrease of 3.84 points) and backward deletion (*P* < 0.01, OFV increase of 6.64 points) was performed. Reduced interindividual and residual variabilities, precision of the parameter estimates, diagnostic plots, and shrinkage were also considered. The extent of shrinkage, as a measure of model overparameterization, was calculated for each PK parameter with the associated IIV.

The final model was validated using the bootstrap method (*n* = 5,000 runs) and prediction-corrected visual predictive checks (pc-VPCs). The median and 95% CIs for the bootstrap results were compared with the estimated PK parameters from the final model. Using pc-VPCs, 1,000 data sets were simulated from the final model parameter estimates, and the 95% confidence intervals (CIs) for the 5th, 50th, and 95th percentiles based on the simulated data sets were calculated and overlaid with the prediction-corrected observed concentration for visual inspection (27).

### Monte Carlo simulations.

Monte Carlo simulations (*n* = 1,000) were performed in R v.3.6.0 with the mlxR package for segmented CrCL levels (20 to 150 mL/min) in four subgroups: group 1, concomitant use of ECMO and CVVHDF; group 2, receiving ECMO only; group 3, weaned from ECMO and receiving CVVHD; group 4, weaned from ECMO and not receiving CVVHDF. The dosing regimen was created based on a total daily piperacillin-tazobactam dose of 24/3, 20/2.5, 16/2, 12/1.5, 9/1.125, or 8/1 g, with an interdose interval of 6 or 8 h and an infusion duration of 0.5 h (intermittent bolus), 3 h or 4 h (extended infusion duration equal to half of the dosing interval), and continuous infusion. Free piperacillin and tazobactam concentrations were calculated by assuming 30% protein binding (28). The PD target for piperacillin was set to a 100% *f*T_>MIC_ of 16 mg/L, which is the clinical breakpoint for P. aeruginosa defined by the European Committee on Antimicrobial Susceptibility Testing (http://www.eucast.org/clinical_breakpoints/) (25), and a probability of target attainment (PTA) of ≥90% was considered optimal (10, 29). In addition, PTAs for 100% *f*T_>MIC_ (MIC of 2, 4, 8, 16, 32, and 64 mg/L) with continuous infusion method were determined to improve usefulness in cases where the MIC in the actual culture could be established. Because tazobactam has only bacteriostatic activity, the proportion of dosing interval above the minimum effective concentration (MEC) was calculated. The PTA for 63% *f*T > 2 mg/L of free tazobactam was evaluated, and the acceptable PTA level was set at ≥50% (30, 31).

### Data availability.

The data sets generated and/or analyzed during the current study are not publicly available, owing to privacy concerns and institutional policies, but are available from the corresponding author on reasonable request.
