# Supplementary material for: Population Pharmacokinetics and Dosing Optimization of Piperacillin-Tazobactam in Critically Ill Patients on Extracorporeal Membrane Oxygenation and the Influence of Concomitant Renal Replacement Therapy
Source: Microbiol Spectr. 2021 Dec 22;9(3):e00633-21. doi: 10.1128/Spectrum.00633-21 (PMC8694146; doi:10.1128/Spectrum.00633-21)
Supplement: SUPPLEMENTAL FILE 1 — Supplemental material. Download SPECTRUM00633-21_Supp_1_seq1.pdf, PDF file, 0.4 MB [file spectrum00633-21_supp_1_seq1.pdf]

Table S1. Piperacillin covariate OFV

Analysis type	Covariate added	Objective functions
**Univariate screening**		
**Covariate for CL**	**GFR**	**–10.3**
	**BUN**	**–8.3**
	**sCr**	**–9.1**
	**eCrCL**	**–20.1**
	**CVVHDF**	**–8**
	**ECMO**	**–11.6**
**Covariate for V1**	**BMI**	**–4.6**
	**T.bili**	**–10.5**
	**CVVHDF**	**–9.6**
	**ECMO**	**–5.2**
**Stepwise covariate modeling**		
**Forward inclusion**		
**Step 1, eCrCL for CL**	**GFR for CL**	**–3.1**
	**BUN for CL**	**+0.2**
	**sCr for CL**	**–2.3**
	**CVVHDF for CL**	**–5.3**
	**ECMO for CL**	**–16.2**
	**BMI for V1**	**–3.1**
	**T.bili for V1**	**–15.1**
	**CVVHDF for V1**	**–12.3**
	**ECMO for V1**	**–1.3**
**Step 2, eCrCL and ECMO for CL**	**CVVHDF for CL**	**–3.2**
	**T.bili for V1**	**–4.9**
	**CVVHDF for V1**	**–8.2**
**Step 3, eCrCL and ECMO for CL & CVVHDF for V1**	**T.bili for V1**	**–5.2**
**Backward deletion**		
**eCrCL and ECMO for CL & CVVHDF and T.bili for V1**	**eCrCL for CL**	**+30.1**
	**ECMO for CL**	**+15.2**
	**CVVHDF for V1**	**+11.2**
	**T.bilifor V1**	**+6.2**
**CrCL and ECMO for CL & CVVHDF for V1**	**eCrCL for CL**	**+31.2**
	**ECMO for CL**	**+16.3**
	**CVVHDF for V1**	**+13.1**

GFR, glomerular filtration rate; BUN, blood urea nitrogen; sCR, serum creatinine; T.bili, total bilirubin; eCrCL, estimated creatinine clearance; ECMO, extracorporeal membrane oxygenators; CVVHDF continuous venovenous hemodiafiltration

Table S2. Tazobactam covariate OFV

Analysis type	Covariate added	Objective functions
**Univariate screening**		
**Covariate for CL**	**GFR**	**–7.1**
	**BUN**	**–5.3**
	**sCr**	**–9**
	**eCrCL**	**–15.2**
	**ECMO**	**–11.6**
**Covariate for V1**	**ECMO**	**–5.3**
**Stepwise covariate modeling**		
**Forward inclusion**		
**Step 1, eCrCL for CL**	**GFR for CL**	**–1.2**
	**BUN for CL**	**–0.8**
	**sCr for CL**	**–0.3**
	**ECMO for CL**	**–18.3**
	**ECMO for V1**	**–2.1**
**Backward deletion**		
**eCrCL and ECMO for CL**	**eCrCL for CL**	**+21.3**
	**ECMO for CL**	**+12.1**

GFR, glomerular filtration rate; BUN, blood urea nitrogen; sCR, serum creatinine; eCrCL, estimated creatinine clearance; ECMO, extracorporeal membrane oxygenators

Table S3. Simulated PTAs for piperacillin (target: 100% fT > MIC of 16 mg/L) in four patient groups

		ECMO on CVVHDF on							ECMO on CVVHDF off						
Total daily dose	Regimen / CrCL (mL/min)	20	40	60	90	110	130	150	20	40	60	90	110	130	150
8g	2g q6h IB	70.3	25.4	5.7	0.3	0	0	0	57.5	13.1	1.6	0	0	0	0
	2g q6h EI	77.3	40.4	13.8	1.5	0.6	0.2	0	73	29.3	7.3	0.8	0.1	0	0
	2g q6h CI	83.8	63.2	34.7	11.1	4.7	2.3	1.6	85.3	64.1	35.2	11.3	4.9	2.4	1.5
9g	3g q8h IB	66.4	20.7	4.4	0.4	0	0	0	50.5	9.2	1	0.1	0	0	0
	3g q8h EI	78.7	41.4	13.1	1.7	0.4	0.2	0	71.6	26.4	6.1	0.3	0.1	0	0
	3g q8h CI	88.6	71.5	48.7	18.5	7.8	3.6	2.3	89.1	72	49.1	18.7	7.9	3.6	2.3
12g	3g q6h IB	87.7	56.1	22.2	3.5	0.9	0.2	0	80.3	38	9.7	1	0	0.1	0
	3g q6h EI	91.9	71.6	41.7	11.5	4.3	1.3	0.7	89.6	59.4	26.8	4.3	0.9	0.1	0.1
	4g q8h IB	81	40.6	12.2	1.2	0.2	0.1	0	66.1	19.6	4	0.2	0.1	0	0
	4g q8h EI	89.2	60.7	29.8	5.7	1.9	0.9	0.1	84.2	47.1	14.9	1.5	0.1	0	0.1
	4g q8h CI	94	85.2	71.6	45.8	27.3	16.6	9.6	94.3	85.5	71.9	45.9	27.5	16.6	9.6
16g	4g q6h IB	93.3	73.3	42.2	10.5	3.6	1.1	0.4	88.3	55.9	20.7	3.6	1.1	0.1	0.1
	4g q6h EI	95.4	85	61.4	26.5	12.9	5.4	2.4	94.1	78	47.5	12.6	4.4	1.2	0.2
	4g q6h CI	96.3	92.6	85.3	69.6	56.5	42.8	29.2	96.4	92.6	85.4	69.7	56.7	42.8	29.2
20g	5g q6h IB	95.8	81.7	56.1	20.9	7.9	3.5	1.3	92.7	66.3	34.5	7.1	2.3	0.8	0.1
	5g q6h EI	96.7	90.9	76.2	42.6	23	12	5.4	96.2	85.6	60.9	23.5	10.5	3.7	1.2
	5g q6h CI	97.1	95.1	91.4	81.4	73.1	63.8	52.8	97.3	95.2	91.4	81.5	73.1	63.9	52.9
24g	6g q6h IB	97	87.1	65.3	29.5	13.8	6.1	2.9	95	76.8	45	12	4.4	1.5	0.4
	6g q6h EI	97.5	93.6	83.6	54.9	35.3	19.4	10.3	97.3	89.5	71.7	35.5	16.4	7.6	3.3
	8g q8h IB	94.9	77.4	47.6	13.6	5.6	2	0.8	89.2	57.4	23.7	4.8	1.2	0.3	0.1

	8g q8h EI	97.3	90.6	73.5	40	20.9	10	4.5	96.1	83.7	55.9	18.7	8.1	2.9	1
	8g q8h CI	98	96.4	94.3	88.1	82	75.6	66.9	98	96.5	94.3	88.1	82	75.6	66.9
		ECMO off CVVHDF on							ECMO off CVVHDF off						
		20	40	60	90	110	130	150	20	40	60	90	110	130	150
8g	2g q6h IB	52.3	14.8	2.8	0.1	0	0	0	37.8	7	0.6	0	0	0	0
	2g q6h EI	63.9	28.5	8.4	0.9	0.1	0.1	0	55.3	17.4	3.9	0.4	0	0	0
	2g q6h CI	76.9	52.9	25.9	7.1	3.2	1.8	1.1	77.8	53.6	26.3	7.2	3.2	1.9	1.3
9g	3g q8h IB	48.4	12.2	2.1	0	0	0	0	28.5	4.6	0.3	0.1	0	0	0
	3g q8h EI	65.4	27.4	8.1	0.9	0.2	0	0	53.2	14.3	3	0	0.1	0	0
	3g q8h CI	81.8	63.6	38	13.7	5.7	3.3	1.9	82.6	64.1	38.1	13.9	5.7	3.3	1.9
12g	3g q6h IB	77.9	42.6	14.9	2.2	0.5	0.1	0	63.2	22.8	5.9	0.4	0	0.1	0
	3g q6h EI	85.6	59.1	31.9	7.7	2.8	1.3	0.5	81.1	46.7	16.8	2.2	0.6	0.1	0.1
	4g q8h IB	66.2	26.8	6.8	0.8	0	0.1	0	47.9	11.1	2.2	0.1	0.1	0	0
	4g q8h EI	80.4	49.6	20.6	3.9	0.9	0.4	0.2	73	31.8	9.8	0.9	0.1	0.1	0
	4g q8h CI	91.2	79.9	65.6	37.4	22.2	13.7	7	91.5	80.1	65.8	37.5	22.2	13.7	7
16g	4g q6h IB	87.7	60.6	32.2	7.2	2.6	0.8	0.2	79.6	41.6	13.8	2.1	0.4	0.1	0
	4g q6h EI	93	78	52.5	19.9	8.6	3.8	1.3	89.8	65.3	36.2	9.1	2.5	0.6	0.1
	4g q6h CI	95.1	90.4	81.5	65.3	52.2	36.7	24.3	95.2	90.5	81.6	65.5	52.3	36.7	24.3
20g	5g q6h IB	92.5	73.3	45.8	14.8	5.7	2.3	0.9	86.2	54.3	23.3	5.1	1.2	0.3	0.1
	5g q6h EI	95.2	86.5	66.9	35.7	18	8.7	3.6	93.4	79.3	52	16.9	6.9	2.3	0.7
	5g q6h CI	96.4	94	89.6	78.2	69.5	59.3	49.1	96.5	94.1	89.7	78.2	69.5	59.4	49.2
24g	6g q6h IB	94.9	80.5	56.2	23.2	10	4.5	1.8	89.5	63.7	33.5	8.7	3	1	0.3
	6g q6h EI	96.7	91.4	77	46.4	29.5	15.9	7.4	95.6	85.1	62.5	26.2	12.8	5.6	2
	8g q8h IB	90.7	66	37.2	9.9	3.9	1.6	0.8	80.9	43.5	15.8	2.9	0.9	0.2	0.1
	8g q8h EI	95.3	85.9	64.7	31.8	16.4	7.5	3.4	92.8	74.5	46	13.9	5.5	1.9	0.6

8g q8h CI	97.3	95.6	93.2	86.1	79.1	72.7	65	97.4	95.6	93.3	86.1	79.1	72.7	65

PTAs above 90% are highlighted in yellow. PTA, probability of target attainment; ECMO, extracorporeal membrane oxygenation; CVVHDF, continuous venovenous hemodiafiltration; CrCL, creatinine clearance; IB, intermittent bolus; EI, extended infusion; CI, continuous infusion

Figures

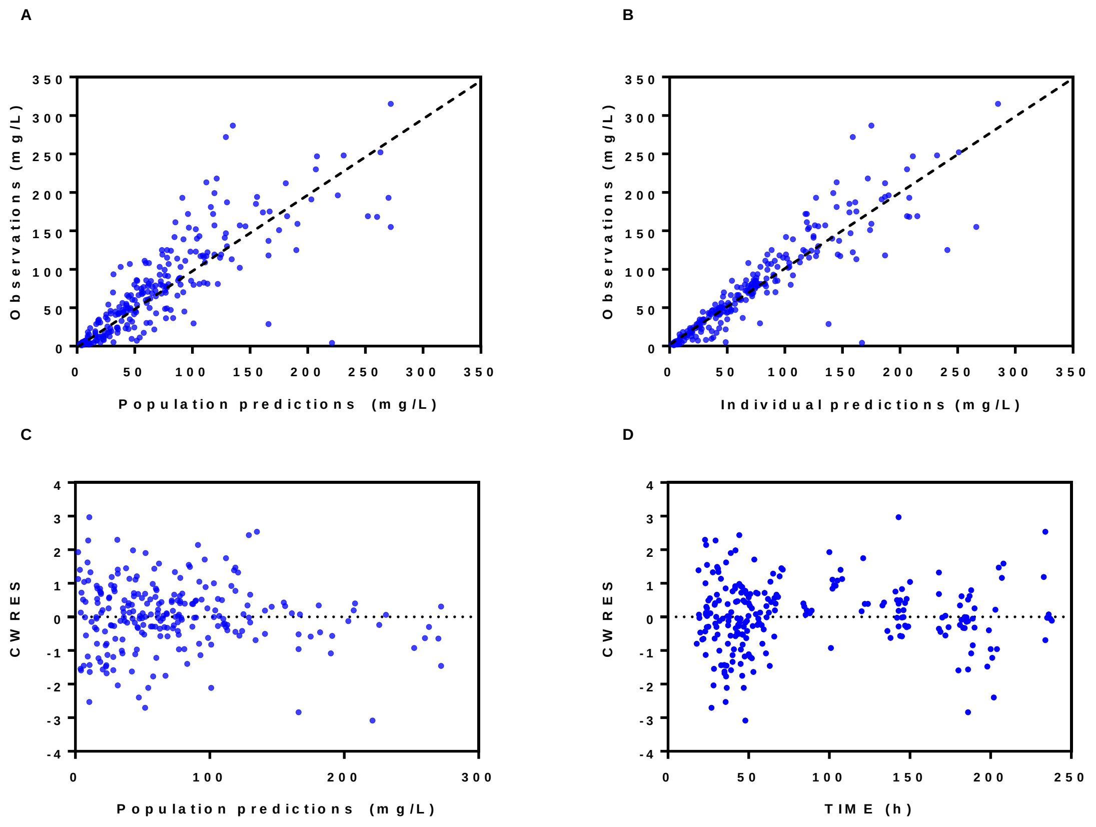

Figure S1. Goodness-of-fit plots for the final population pharmacokinetic model of piperacillin. Observed piperacillin concentrations versus population predicted concentrations (A) and individual predicted concentrations (B), and conditional weighted residuals (CWRES) versus population predicted concentrations (C) and time (D).

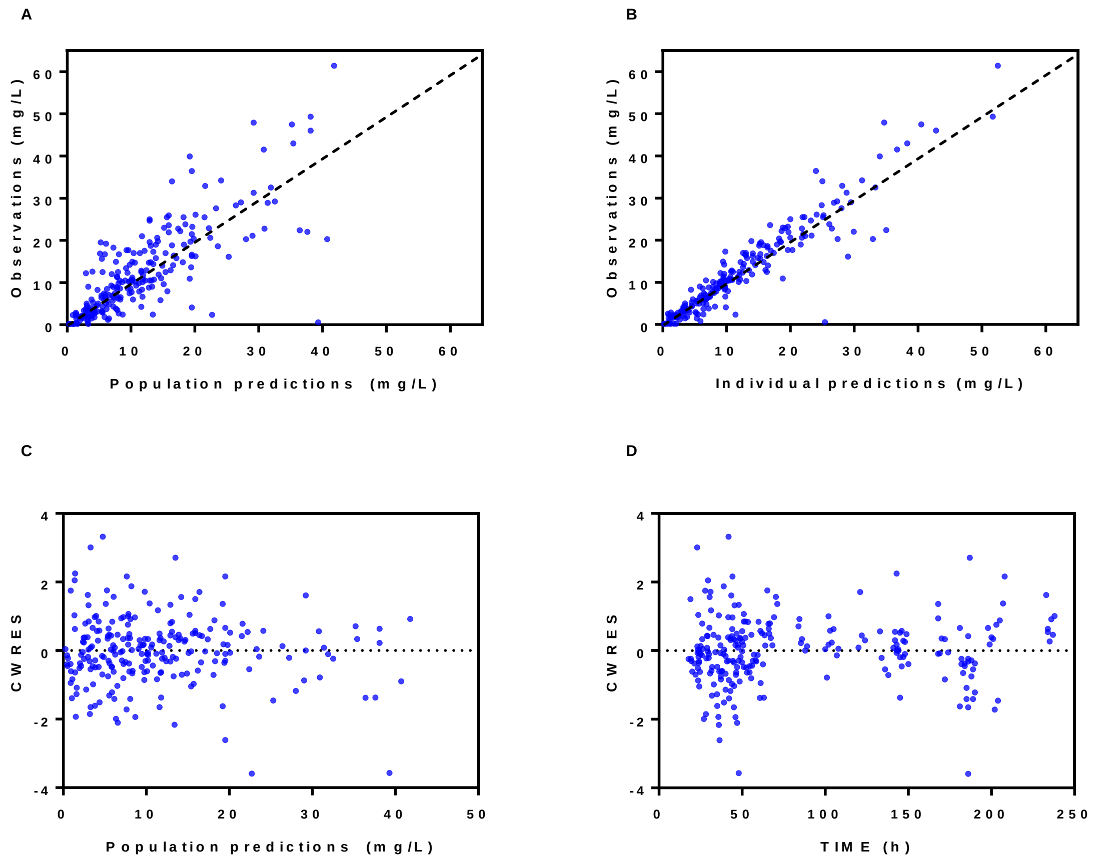

Figure S2. Goodness-of-fit plots for the final population pharmacokinetic model of tazobactam. Observed tazobactam concentrations versus population predicted concentrations (A) and individual predicted concentrations (B), and conditional weighted residuals (CWRES) versus population predicted concentrations (C) and time (D).

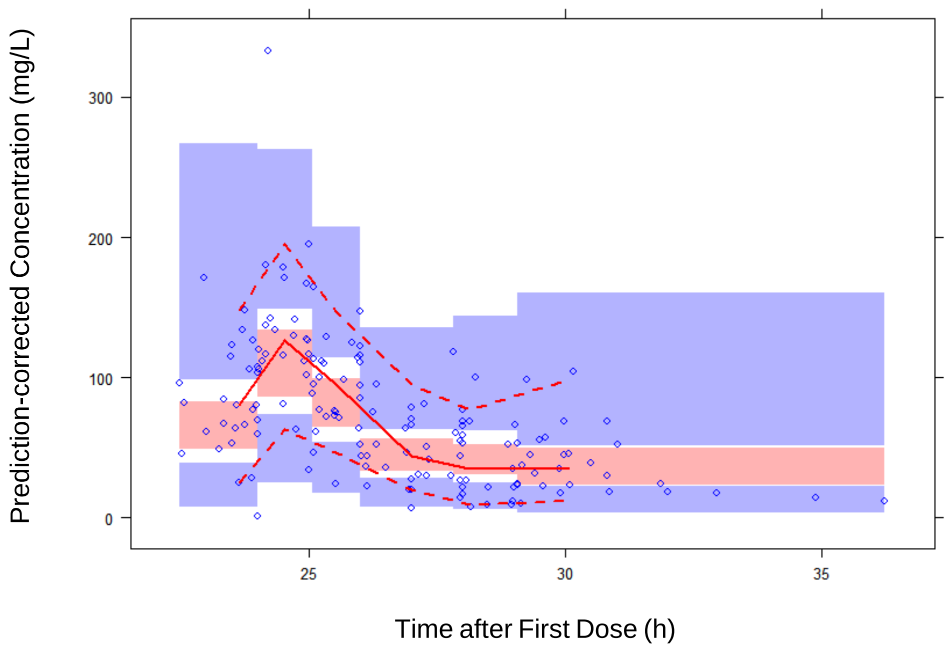

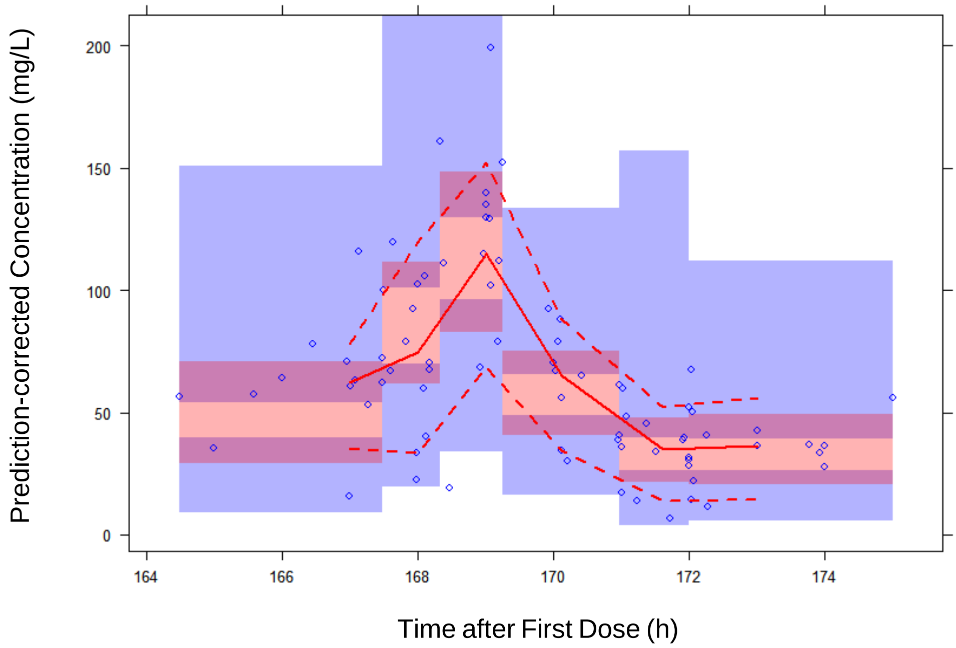

Figure S3. **Prediction-corrected visual predictive checks (pc-VPCs) of the piperacillin final population pharmacokinetic model.** The plots correspond to 'on ECMO' (upper panel) and 'off ECMO' (lower panel). Open circles, observed piperacillin concentrations; solid line, median; lower and upper dashed lines, 5th and 95th percentiles of the simulated data, respectively; shaded areas, 95% confidence intervals for simulated predicted median, 5th percentile, and 95th percentile constructed from 1,000 simulated datasets of individuals from the original dataset.

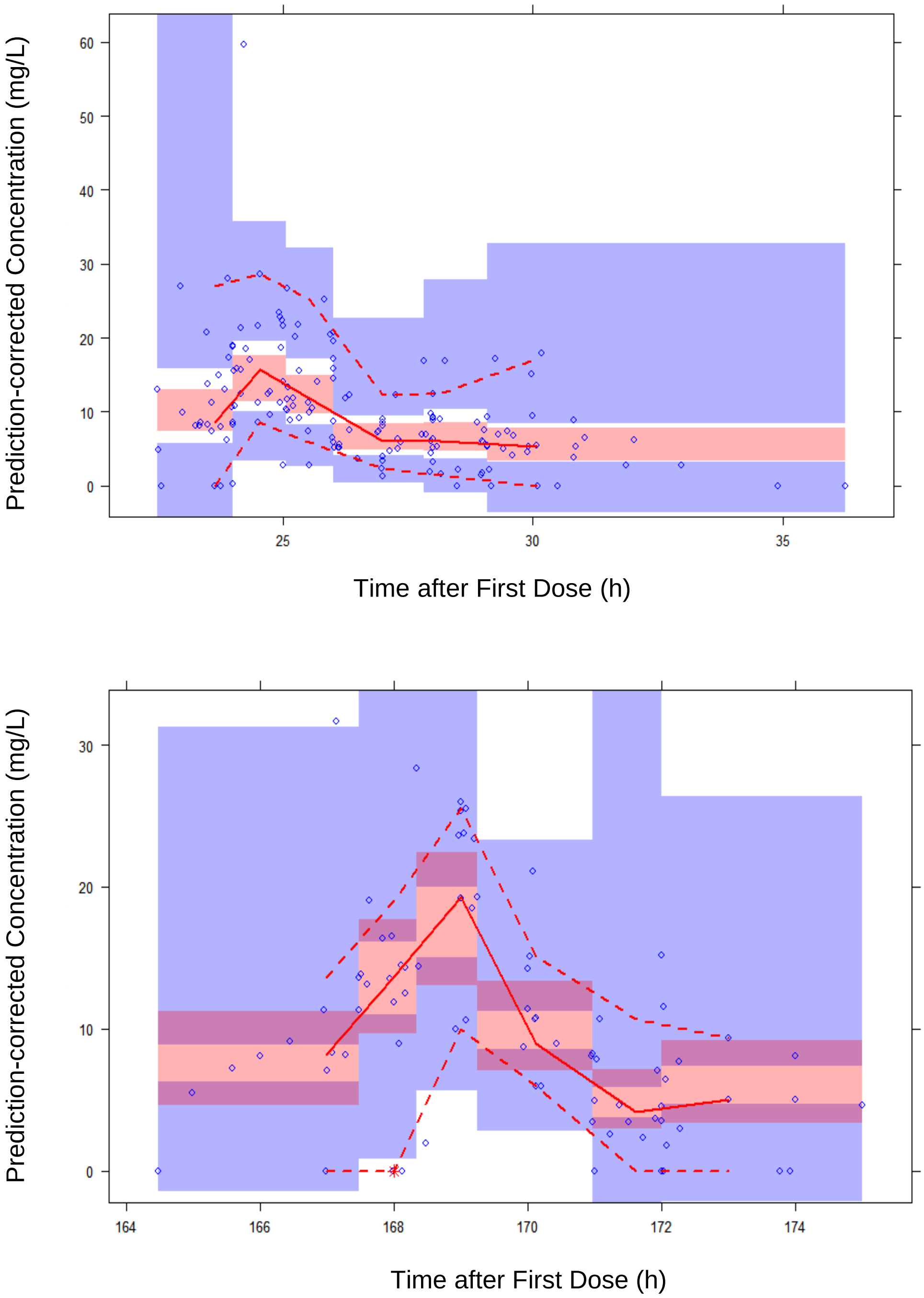

Figure S4. Prediction-corrected visual predictive checks (pc-VPCs) of the tazobactam final population pharmacokinetic model. The plots correspond to 'on ECMO' (upper panel) and 'off ECMO' (lower panel). Open circles, observed tazobactam concentrations; solid line, median; lower and upper dashed lines, 5th and 95th percentiles of the simulated data, respectively; shaded areas, 95% confidence intervals for simulated predicted median, 5th percentile, and 95th percentile constructed from 1,000 simulated datasets of individuals from the original dataset.
